# Neurobiological Implications of Chronic Stress and Metabolic Dysregulation in Inflammatory Bowel Diseases

**DOI:** 10.3390/diseases12090220

**Published:** 2024-09-18

**Authors:** Aleksandar Sic, Kiana Cvetkovic, Eshanika Manchanda, Nebojsa Nick Knezevic

**Affiliations:** 1 Department of Anesthesiology, Advocate Illinois Masonic Medical Center, Chicago, IL 60657, USA; aca.smed01@gmail.com (A.S.); kiana.cvetkovic@gmail.com (K.C.); eshanikam003@gmail.com (E.M.); 2 Faculty of Medicine, University of Belgrade, 11000 Belgrade, Serbia; 3 Department of Anesthesiology, University of Illinois, Chicago, IL 60612, USA; 4 Department of Surgery, University of Illinois, Chicago, IL 60612, USA

**Keywords:** chronic stress, metabolic dysregulation, inflammatory bowel diseases

## Abstract

Chronic stress is a significant factor affecting modern society, with profound implications for both physical and mental health. Central to the stress response is cortisol, a glucocorticoid hormone produced by the adrenal glands. While cortisol release is adaptive in acute stress, prolonged exposure to elevated levels can result in adverse effects. This manuscript explores the neurobiological implications of chronic stress and its impact on metabolic dysregulation, particularly in the context of inflammatory bowel diseases (IBDs). The hypothalamic–pituitary–adrenal (HPA) axis regulates cortisol production, which influences metabolism, immune response, and neurobiology. Elevated cortisol levels are associated with the development and exacerbation of metabolic disorders like IBD and contribute to neurodegenerative processes, including cognitive impairments and increased susceptibility to psychiatric conditions. The interaction between cortisol and its receptors, particularly glucocorticoid receptors, underscores the complexity of these effects. This review aims to elucidate the mechanisms through which chronic stress and cortisol dysregulation impact metabolic health and neurobiological function, providing insights into potential therapeutic strategies for mitigating these effects.

## 1. Introduction

In contemporary society, chronic stress has become an almost unavoidable element of everyday life, exerting profound effects on both physical and mental health [1]. Cortisol, a crucial glucocorticoid hormone produced by the adrenal glands, plays a central role in the body’s response to stress. While the release of cortisol in response to acute stress is essential for adaptive, short-term responses, the persistent elevation of cortisol levels due to chronic stress can lead to a range of detrimental effects on various physiological systems [2].

Chronic stress and elevated cortisol levels have significant implications for metabolic health. Prolonged exposure to high cortisol levels is closely linked to the development of metabolic disorders, including obesity, insulin resistance, and inflammatory bowel diseases (IBDs) [3]. These metabolic disorders often stem from the complex interactions between cortisol’s effects on glucose metabolism, fat distribution, and immune function [4]. For instance, cortisol-induced insulin resistance contributes to abnormal glucose homeostasis, potentially leading to type 2 diabetes [5]. Additionally, chronic stress and elevated cortisol levels can exacerbate inflammation, influencing the onset and progression of IBD by affecting gut permeability and immune responses [3].

The impact of chronic stress and cortisol is not limited to metabolic health; it extends to neurobiology as well. Elevated cortisol levels have been implicated in neurodegenerative processes, neuroinflammation, and structural changes in the brain. Chronic exposure to high cortisol levels can lead to hippocampal atrophy, which is associated with cognitive impairments and an increased susceptibility to psychiatric disorders such as depression and anxiety [6,7,8]. Understanding these neurobiological changes is crucial for developing targeted therapeutic strategies to mitigate the cognitive and emotional effects of chronic stress, such as interventions aimed at reducing hippocampal atrophy, modulating neuroinflammation, and addressing the dysregulation of the hypothalamic–pituitary–adrenal (HPA) axis [9,10,11].

The aim of this narrative review is to provide a comprehensive synthesis of the neurobiological implications of chronic stress and metabolic dysregulation in IBD. This review seeks to integrate and analyze the current literature to elucidate the role of chronic stress and elevated cortisol levels in influencing metabolic pathways and neurobiological health within the context of IBD. We aim to provide a thorough overview of how chronic stress exacerbates metabolic disturbances and contributes to neurobiological alterations in IBD patients, drawing from both animal model studies and clinical observations. Our objective is to summarize key findings regarding the interactions between stress, metabolism, and neurobiology and to clarify how these interactions impact disease progression and symptomatology. By highlighting existing research and conceptual frameworks, this review aims to enhance the understanding of the multifaceted effects of chronic stress on IBD, offering insights that can inform clinical practice and guide future research directions in this field.

Furthermore, we will review current therapeutic approaches and propose potential pathways for future interventions to manage and alleviate the adverse effects of chronic stress.

## 2. Mechanisms of Cortisol Regulation and Physiological Effects

Cortisol is a steroid hormone that plays a key role in regulating many processes in the body, including metabolism, immune response, and stress reaction [12,13]. Cortisol release adheres to the circadian rhythm, controlled by the internal clock located in the suprachiasmatic nucleus [14,15]. It is produced in the adrenal glands and controlled by the HPA axis [16].

The HPA axis is a central stress response system crucial for regulating cortisol, the body’s primary glucocorticoid hormone. When a stressor is perceived, the hypothalamus releases corticotropin-releasing hormone (CRH) from the paraventricular nucleus into the hypophyseal portal system. CRH then stimulates the anterior pituitary gland to secrete adrenocorticotropic hormone (ACTH). ACTH travels through the bloodstream to the adrenal cortex, where it binds to melanocortin receptors, stimulating the production and release of cortisol [16]. In addition to cortisol, chronic stress triggers the release of other key hormones and neurotransmitters that contribute to the stress response and its physiological effects. Adrenaline and norepinephrine, released by the adrenal medulla, play a crucial role in the acute stress response, facilitating immediate physical reactions—such as increased heart rate and energy mobilization. However, their chronic elevation can lead to sustained cardiovascular strain and exacerbate stress-related conditions [17]. Neurotransmitters like serotonin, dopamine, and gamma-aminobutyric acid (GABA) are also heavily influenced by stress. Disruptions in these systems can contribute to mood disorders, including depression and anxiety, by altering mood regulation, motivation, and stress resilience [18]. Furthermore, chronic stress induces the release of proinflammatory cytokines, including interleukin-6 (IL-6) and tumor necrosis factor-alpha (TNF-α), which contribute to systemic inflammation. This inflammation can worsen metabolic disorders and promote neurodegenerative processes, highlighting the interconnectedness of stress, immune function, and overall health [19].

Cortisol exerts multiple physiological effects: it increases blood glucose levels by promoting gluconeogenesis and inhibiting glucose uptake in peripheral tissues, modulates fat and protein metabolism, and possesses potent anti-inflammatory and immunosuppressive properties. Additionally, cortisol influences cardiovascular function by maintaining blood pressure and impacts the brain, affecting mood, cognition, and memory. This hormone operates in a feedback loop; elevated cortisol levels inhibit CRH and ACTH secretion, thus preventing excessive cortisol production. Chronic stress can disrupt this delicate balance, leading to persistently high cortisol levels, which are associated with numerous health issues, including metabolic disorders like type 2 diabetes, cardiovascular diseases such as hypertension, and psychiatric conditions like depression and anxiety. Understanding the intricate workings of the HPA axis and its regulation of cortisol is essential for developing strategies to manage stress and mitigate its adverse health impacts [20,21].

**Figure 1:**
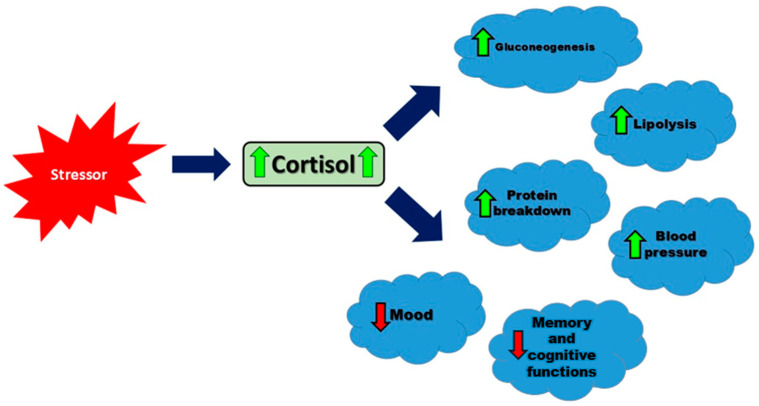
The main physiological responses to cortisol secretion in response to a stressor. Arrows represent the direction of influence: green solid arrows indicate an increase in the target function, while red dashed arrows indicate a decrease.

ACTH regulates multiple steps in the steroidogenesis pathway, such as enhancing low-density lipoprotein (LDL) receptor expression and converting cholesterol to pregnenolone, the initial and rate-limiting step in cortisol production [22]. LDL receptors (LDL-Rs) are fundamental for the uptake of cholesterol into cells for steroid hormone synthesis. Data have indicated that ACTH upregulates LDL receptor (LDL-R) activity on adrenal cortical cells, ensuring efficient cholesterol uptake from the bloodstream for steroidogenesis [23]. Once in the cell, cholesterol is transported to the mitochondria for conversion to pregnenolone. StAR is a key regulatory protein that transports cholesterol to the mitochondria. ACTH activates a cAMP-PKA signaling pathway to stimulate StAR activity, allowing for the uptake of cholesterol and conversion to pregnenolone. Through the initiation and maintenance of the steroidogenic process, ACTH plays a critical role in producing sufficient levels of cortisol for physiological needs [23,24]. Cortisol is released in a circadian rhythm, with the highest levels in the morning and the lowest in the evening [14]. This hormone increases blood glucose levels, affects fat and protein metabolism, has anti-inflammatory and immunosuppressive properties, helps maintain blood pressure and blood volume, and impacts brain functions, including mood and memory [25]. Understanding these multifaceted effects of cortisol is crucial, as illustrated in Figure 1, which summarizes the key physiological responses to cortisol secretion.

Cortisol exists in the body in two forms: free (unbound) and bound. The free form of cortisol is biologically active, able to penetrate cells and bind to glucocorticoid receptors inside them, thereby initiating its effects on metabolism, immune response, and stress reaction. The bound form of cortisol attaches to proteins like albumin and cortisol-binding globulin (CBG), serving as a reservoir that helps maintain a stable cortisol level in the blood. This binding also extends cortisol’s half-life in circulation, preventing rapid fluctuations and enabling the body to better manage stressful situations [26,27]. In the steady state, there is a balance between the two forms, with 30–35% of CBG in the low-affinity form [28]. Both forms are essential for proper bodily function; the free form is critical for immediate action, while the bound form ensures the stability and prolonged availability of cortisol [26,27].

Cortisol plays a vital role in the body’s adaptive response to stressful situations. It aids in energy mobilization, regulates the immune response, and helps maintain homeostasis. However, prolonged or excessive exposure to cortisol can have a wide range of effects on the endocrine system. One of the key mechanisms is cortisol’s ability to inhibit the secretion of other hormones, particularly growth hormone (GH) and sex hormones such as testosterone and estrogen [29]. High concentrations of cortisol in the blood during stress episodes suppress the release of growth hormone from the pituitary gland. This can have far-reaching consequences, including reduced tissue growth and regeneration, which is particularly significant in children and adolescents [30]. Additionally, elevated cortisol levels can affect the function of the reproductive system. In males, high cortisol levels can lower testosterone levels, leading to decreased libido, reduced sperm count, and decreased muscle mass. In females, high cortisol levels can disrupt the menstrual cycle and reduce fertility [31].

These effects illustrate how cortisol, while crucial for short-term adaptation to stress, can have detrimental consequences when present in elevated levels or over a prolonged period. This underscores the importance of balancing its secretion to maintain the health of the endocrine system and overall well-being [32]. Cortisol exerts its effects through glucocorticoid receptors (GRs), which are widely distributed throughout the body, including in almost every cell type. GRs belong to the nuclear receptor superfamily and act as transcription factors that regulate gene expression in response to cortisol binding [33]. Upon entering cells, cortisol binds to cytoplasmic GRs, leading to a conformational change that exposes nuclear localization signals. This complex then translocates into the cell nucleus, where it binds to specific DNA sequences known as glucocorticoid response elements (GREs). By binding to GREs, the cortisol–GR complex modulates the transcription of target genes, either enhancing or suppressing their expression depending on the context [34].

The actions of cortisol via GRs are diverse and influence a wide range of physiological processes. For example, in metabolic regulation, cortisol stimulates gluconeogenesis and inhibits glucose uptake in peripheral tissues, thereby increasing blood glucose levels during stress. In immune function, cortisol suppresses inflammatory responses by inhibiting the transcription of proinflammatory cytokines. Additionally, cortisol plays a crucial role in modulating brain function, affecting mood, memory, and behavior through its actions on neurons and glial cells in the central nervous system [35]. The sensitivity and responsiveness of GRs to cortisol vary among tissues and cell types, reflecting the complex regulatory mechanisms that ensure precise physiological responses to changing environmental and internal conditions. The dysregulation of cortisol–GR signaling, such as chronic exposure to elevated cortisol levels, can lead to pathological conditions, including metabolic disorders, immune dysfunction, and psychiatric disorders [36,37]. Understanding the intricate interactions between cortisol and its receptors provides insights into how these systems contribute to both health and disease, highlighting the importance of maintaining balanced cortisol signaling for optimal physiological function [27].

The dysregulation of cortisol, a hormone crucial for regulating numerous physiological processes, can lead to significant clinical implications. This includes Cushing’s syndrome, characterized by excessive cortisol production, which highlights the detrimental effects of hypercortisolism such as central obesity, hypertension, osteoporosis, and glucose intolerance [38]. Cushing’s Disease, a specific form of Cushing’s syndrome, is caused by a pituitary microadenoma that leads to excess cortisol production. This condition results in hypertension, hyperglycemia, excess fat deposition, and other complications. Pituitary hyperplasia causes an increased production of ACTH, which ultimately results in cortisol overproduction by the adrenal glands. Screening for elevated cortisol levels in patients with potential Cushing’s Disease can be performed using salivary cortisol, urinary free cortisol, or by observing suppressed cortisol levels after a dexamethasone dose to rule out exogenous steroid use [39]. Conversely, adrenal insufficiency, as seen in conditions like Addison’s disease, results from insufficient cortisol and aldosterone production, leading to symptoms such as fatigue, weight loss, low blood pressure, and skin hyperpigmentation. Disorders affecting the HPA axis can further disrupt cortisol regulation, contributing to conditions like chronic stress-related disorders, depression, and anxiety. Understanding these clinical implications underscores the importance of maintaining a balanced cortisol level for overall health and well-being [40].

Furthermore, cortisol plays a pivotal role in regulating inflammation through a complex mechanism involving the body’s immune response. As the primary glucocorticoid, cortisol is released in response to stressful situations, activating its receptors in target tissues. Within the immune system, cortisol suppresses the activity of proinflammatory cytokines, such as interleukin-1 (IL-1), IL-6, TNF-α, interleukin-12 (IL-12), interleukin-18 (IL-18), interferon-gamma (IFN-γ), interleukin-17 (IL-17), and interleukin-23 (IL-23), while promoting anti-inflammatory cytokines such as interleukin-10 (IL-10), interleukin-4 (IL-4), interleukin-13 (IL-13), interleukin-1 receptor antagonist (IL-1Ra), and transforming growth factor-beta (TGF-β). This hormonal mechanism reduces the reactivity of the immune system, which is beneficial in the short term for controlling acute inflammation. However, prolonged exposure to elevated cortisol levels, such as in chronic stress, can suppress immune responses and increase the risk of inflammatory diseases. Therefore, a balanced regulation of cortisol is crucial for maintaining immune homeostasis and overall health [41].

**Figure 2:**
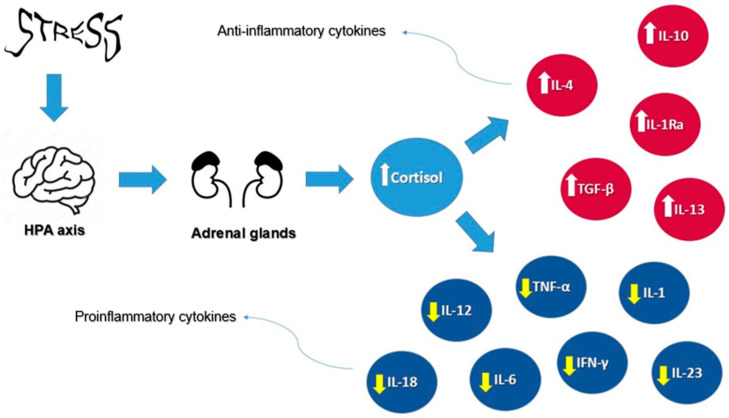
Cortisol’s effects on cytokines under stress conditions. Arrows represent the direction of influence: white arrows indicate an increase in anti-inflammatory cytokines, while yellow arrows indicate a decrease in pro-inflammatory cytokines.

Figure 2 illustrates the effects of cortisol on cytokines under stress conditions, highlighting its dual role in modulating proinflammatory cytokines, such as IL-1, IL-6, and TNF-α, while promoting anti-inflammatory cytokines such as IL-10 and IL-4.

## 3. Animal Models in Cortisol Research

Animal models are crucial for understanding the role of cortisol in stress, enabling research into the neurobiological mechanisms and consequences of chronic stress [42]. Studies in adult mice showed that ten days of chronic stress led to lasting behavioral changes, including increased anxiety and depressive symptoms. In male rats, chronic stress induces dendritic atrophy in CA3 pyramidal neurons, whereas females do not exhibit these changes, indicating sex differences in stress response. Adolescent stress in rats can increase susceptibility to schizophrenia due to alterations in prefrontal cortex function. Additionally, adolescent stress causes enduring behavioral changes such as increased anxiety and depressive symptoms, as well as changes in HPA axis function and the hippocampus. These findings provide crucial insights into the potential mechanisms through which chronic stress and elevated cortisol levels may contribute to the development of various mental disorders including depression, anxiety, and schizophrenia, as well as the role of sex differences in these processes [43].

### 3.1. Comparison of Corticosterone and Cortisol Levels

Rodents typically have higher levels of corticosterone compared to cortisol, with cortisol levels usually being less than 1% of corticosterone levels [44]. Studies involving mice and hamsters used different methods (EIA and RIA) to measure corticosterone and cortisol levels in response to various stressors. In mice subjected to acute stress (48 h of movement restriction and forced swimming), cortisol levels rose earlier and remained elevated longer than corticosterone levels. However, during chronic stress (8 h of movement restriction daily for 23 days), corticosterone levels decreased significantly from the first day onward, while cortisol levels remained stable [45]. Similarly, in hamsters, following both acute and chronic stress, corticosterone levels initially exceeded cortisol levels. Following acute stress, both hormones increased, but corticosterone levels were consistently higher. In contrast, after chronic stress, only cortisol levels showed a significant increase [46]. These findings underscore that corticosterone and cortisol are regulated independently and respond differently to stressors, potentially due to their varying sensitivity to hormones like ACTH across different species. This variability highlights the importance of measuring both hormones to fully understand adrenal function in rodents [47].

### 3.2. Implications for Research and Interventions

By comprehending how cortisol levels are intricately regulated in response to stress, scientists can devise specific interventions and therapies for stress-related disorders in both animals and humans. This understanding enables tailored approaches to mitigate the effects of stress on health and well-being [48].

## 4. Measurements

An accurate measurement of cortisol levels is crucial for understanding its physiological impact and diagnosing related disorders. Different biological samples—blood, saliva, urine, and hair—offer unique insights into cortisol dynamics and its relevance in various contexts [49].

Blood measurements (serum or plasma assays) provide immediate levels, making them essential in clinical settings for diagnosing adrenal disorders such as Cushing’s syndrome or adrenal insufficiency [50]. They allow healthcare providers to assess acute cortisol levels and monitor treatment responses. Advances in technology have improved assay sensitivity, allowing for precise measurements even at lower concentrations, which is useful for detecting subtle hormonal fluctuations in patients with milder adrenal dysfunction [51].

Saliva sampling offers a non-invasive method for measuring free cortisol, which is useful for assessing diurnal variations and chronic stress levels [52]. Salivary cortisol reflects the free, unbound form of cortisol, providing an accurate representation of bodily levels. The natural diurnal rhythm of cortisol typically peaks in the early morning but may fluctuate throughout the day with chronic stress. By assessing cortisol levels throughout the day using saliva sampling, it presents an opportunity to capture variations in cortisol missed in a single blood sample. There are guidelines that involve a series of methodical steps that ensures accurate collection and measurement. The process begins with pre-collection guidelines including no eating or drinking alcohol prior, no flossing to avoid bleeds, and rinsing the mouth with water [52]. The collection device includes a swab and storage tube which are then centrifuged and analyzed by tandem mass spectrometry coupled to liquid chromatography [52]. Salivary cortisol is frequently used in research and clinical psychology to evaluate chronic stress levels and understand daily cortisol fluctuations. Recent improvements in collection methods have reduced variability and increased reliability, broadening their application across different populations and settings [53].

Urine samples capture cortisol metabolites over time, providing a broader perspective on long-term cortisol exposure and helping in the detection of conditions like adrenal tumors [54]. Cortisol can be detected separately from other steroid components in the urine, being measured alone or as part of a broad panel of steroid compounds. Certain techniques, such as immunoassays ELISA and RIA, have high specificity to quantify cortisol levels without the interference of other steroids. LC-MS is a comprehensive steroid profiler that can also be used to separate cortisol from other compounds. There are several analytical methods that measure cortisol levels, either as a broader panel or as a separate entity depending on the diagnostic purpose [55]. Advances in analytical techniques have improved the accuracy of cortisol metabolite quantification, enhancing diagnostic capabilities for conditions associated with abnormal cortisol metabolism, such as metabolic syndrome and obesity-related disorders [56].

Hair cortisol analysis reflects cortisol levels over several months and is increasingly used in epidemiological studies and occupational health to assess chronic stress levels [57,58]. Each method has its advantages and is chosen based on the specific research or clinical questions, emphasizing the importance of selecting the appropriate technique for accurate cortisol assessment in diverse settings [49].

## 5. Chronic Stress

Chronic stress occurs when a stressor persists over an extended period, leading to both psychological and psychological effects. This prolonged stress increases the risk of various health problems, both physical and mental. The body’s stress response involves two systems: the Sympathetic–Adreno-Medullar System (SAM axis), which acts quickly, and the HPA axis, which responds more slowly [59].

The SAM axis reacts within minutes of a stressor through the release of neurotransmitters such as noradrenaline, dopamine, and serotonin, particularly in the hippocampus, the amygdala, the prefrontal cortex, and the nucleus accumbens. This activation of the sympathetic nervous system enhances alertness, arousal, cognition, and attention through G protein-coupled receptors and the cyclic adenosine monophosphate (cAMP) signaling pathway [60].

Chronic stress can lead to elevated levels of IL-6 and plasma cortisol, along with decreased levels of cAMP-responsive element-binding protein (CREB) and brain-derived neurotrophic factor (BDNF), similar to changes seen in depression and mood disorders [61]. It causes structural changes in the brain, including the expansion of basolateral amygdala (BIA) neurons, loss of spines, and shrinkage of dendrites in the medial amygdala and prefrontal cortex (PFC) [62]. These changes resemble those found in the brains of postmortem depressed patients [63]. Such alterations are linked to increased anxiety, PTSD-like behaviors, reduced resilience, and impaired memory and learning [64].

Chronic stress affects multiple organ systems, including the cardiovascular, respiratory, immune, and reproductive systems. The sustained activation of the sympathetic nervous system and HPA axis leads to elevated levels of cortisol and epinephrine levels, causing oxidative stress, endothelial dysfunction, and inflammation. This contributes to the development of atherosclerosis and compromised vascular function [51]. Inflammatory events related to stress may account for about 40% of atherosclerotic cases without other known risk factors [65].

Stress can also induce the acute phase response similar to that seen with infections and tissue damage, leading to increased inflammation [56]. Immune suppression from chronic stress heightens susceptibility to respiratory infections, exacerbates conditions like chronic obstructive pulmonary disease (COPD) and asthma [54,58], and can activate latent viruses [62]. Stress may also increase the risk of certain cancers by suppressing Type 1 cytokines and protective T cells while enhancing suppressor T-cell function [66,67].

The loss of negative feedback on the HPA axis resulting in elevated cortisol contributes to elevated inflammatory markers, oxidative stress, and thyroid and sex hormone dysfunction [68]. Glucocorticoids directly inhibit pituitary gonadotropin, growth hormone (GH), and thyroid-stimulating hormone (TSH), thus decreasing reproductive, growth, and thyroid functions [69]. This cortisol dysfunction results in a decrease in luteinizing hormone (LH) and follicle-stimulating hormone (FSH). In women, this impairs ovarian function, leading to menstrual irregularities, anovulation, and infertility. Meanwhile, men have decreased testosterone production, impaired sexual drive, erectile dysfunction, and decreased sperm quality [70].

Acute stress is a short-term physiological response characterized by the body’s immediate reaction to a perceived threat or challenge. This response triggers rapid physiological changes aimed at preparing the body to cope with the stressor. It typically involves the release of stress hormones like adrenaline and noradrenaline, which enhance alertness, sharpen cognitive function, and mobilize energy reserves for quick action [71].

The acute stress response is essential for survival, mobilizing energy reserves and increasing heart rate and blood pressure to prepare for action. It also sharpens focus and temporarily suppresses non-essential bodily functions to prioritize immediate survival needs [72].

Unlike chronic stress, which persists over an extended period and involves the sustained activation of stress pathways like the SAM and HPA axes, acute stress is short-lived and adaptive [73]. It plays a crucial role in responding to sudden threats, such as physical danger or time-limited challenges, and is typically followed by a return to baseline physiological functioning once the threat subsides [74].

The effective management of acute stress involves strategies such as relaxation techniques, deep breathing, and cognitive reframing to mitigate its immediate impact and prevent it from becoming chronic. Recognizing the distinction between acute and chronic stress helps in developing targeted interventions to maintain overall health and well-being in the face of different types of stressors [75].

## 6. Cortisol and Chronic Stress

Chronic stress is a significant precursor to various diseases and health conditions, often leading to poor health outcomes. While acute stress can be beneficial by motivating adaptive responses to immediate challenges, chronic stress has a detrimental effect due to sustained increases in ‘fight or flight’ chemicals. Both acute and chronic stress can be quantitatively assessed through metrics like blood pressure (BP), heart rate (HR), and other measurable changes. However, interpreting physiological and psychological changes requires examining the circulating levels of metabolic hormones. Short-term cortisol changes can be reflected in serum and saliva, while long-term levels can be measured through either circadian variation or protein-binding capacity [76]. Understanding and assessing the body’s stress response are crucial for evaluating the impact of chronic stress on health.

**Figure 3:**
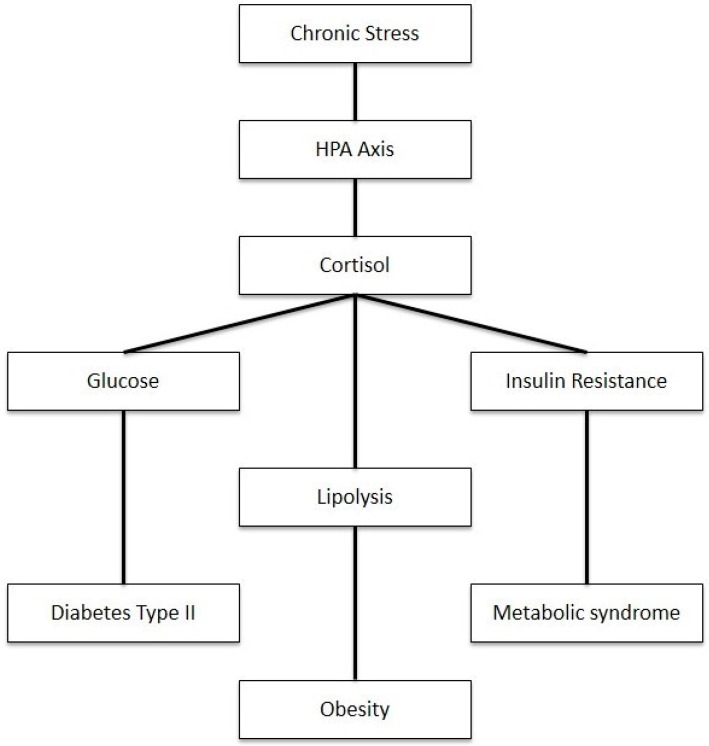
Impact of chronic stress on metabolism through HPA axis activation.

As shown in Figure 3, disruptions in cortisol regulation have significant implications for multiple body systems. The elevation of cortisol can cause a myriad of metabolic disorders such as metabolic syndrome, insulin resistance, and type 2 diabetes. Cortisol is known as an anti-inflammatory that is stimulated by stress. While useful in the short term, prolonged stress intensifies cortisol secretion through repeated surges altering the negative feedback response and regulation of the HPA axis, resulting in cortisol dysfunction [77]. The disinhibition of the HPA axis and elevated cortisol levels stimulate hepatic gluconeogenesis and cause direct and insulin-mediated effects on adipose tissue and skeletal muscle, thus causing increased visceral adipose tissue, insulin resistance, dyslipidemia, and hypertension [78]. Given this, recent studies have shown a correlation between free cortisol levels and subtle changes in HPA activity.

There is a bidirectional relationship between cortisol and insulin. Cortisol stimulates food intake, while insulin inhibits it. This relationship is mediated by the hypothalamic neuropeptide-Y (NPY), which is regulated by cortisol and insulin [79,80]. Chronic exposure to high levels of glucocorticoids inhibits insulin release by binding to glucocorticoid receptors on pancreatic beta cells and decreasing the efficacy of calcium in insulin granule exocytosis [81,82]. Excess cortisol also impairs glucose metabolism by reducing the expression of glucose transporter GLUT-2 and glucokinase, leading to decreased glucose uptake and phosphorylation in pancreatic beta cells [83]. Additionally, excess cortisol can cause a compensatory downregulation or resistance of the glucocorticoid receptor (GR), reducing cortisol binding and contributing to insulin resistance [84]. Therefore, cortisol contributes to decreased insulin sensitivity, which is a significant health concern.

Metabolic syndrome—a cluster of disorders including central obesity, insulin resistance, hypertension, and atherogenic dyslipidemia—is closely linked to chronic stress and cortisol dysregulation [85]. This syndrome is associated with the hyperactivity of the HPA axis, leading to a state of “functional hypercortisolism” [86,87]. Data suggest that while plasma cortisol concentration may be low to normal, urinary free cortisol clearance is increased [79]. Although cortisol plays a major role as an anti-inflammatory hormone, prolonged stress can lead to cortisol dysfunction, resulting in widespread inflammation and pain through elevated inflammatory by-products like free radicals, cellular death, and tissue degeneration [83]. Studies in patients with abdominal obesity have shown HPA axis hyperactivity, with increased urinary cortisol ratios, blood pressure, fasting glucose, and triglycerides. Additionally, mutations or an enhanced expression of the enzyme 11beta-hydroxysteroid dehydrogenase, which converts cortisol to inactive cortisone, may increase the risk of obesity. An enzyme inhibitor, such as Carbenoxolone, may serve as a therapeutic target for obesity and also improve insulin sensitivity [88].

## 7. Metabolic Dysregulation

Metabolic dysregulation plays a crucial role in the pathophysiology of IBD, involving complex interactions between energy metabolism, immune system dysfunction, and endocrine abnormalities [89].

Irritable Bowel Syndrome (IBS) is a complex functional gastrointestinal disorder that manifests with a range of symptoms, including abdominal pain, bloating, and changes in bowel habits such as diarrhea, constipation, or a combination of both. The precise etiology of IBS remains elusive; however, it is widely believed to involve a multifactorial interplay of several mechanisms. These include alterations in gut motility, increased visceral sensitivity, disturbances in gut–brain communication, immune system dysregulation, and psychological factors such as stress and anxiety [90].

The diagnosis of IBS is typically made based on clinical criteria after other gastrointestinal conditions have been excluded. The symptoms of IBS are diverse and can fluctuate, with periods of remission often interspersed with exacerbations triggered by dietary factors, emotional stress, hormonal changes, and environmental influences [91]. The pathophysiology of IBS is intricately linked to disruptions in the gut–brain axis. This axis represents a bidirectional communication network between the gastrointestinal tract and the central nervous system, where any disruption can lead to abnormal gastrointestinal function. Specifically, visceral hypersensitivity is a notable feature of IBS, in which patients exhibit an exaggerated response to normal gut stimuli, leading to significant abdominal pain and discomfort [92,93].

Cortisol, a principal stress hormone, plays a critical role in IBS through its effects on the gastrointestinal system. It acts on receptors located on smooth muscle cells in the gut, influencing motility and transit time. Elevated cortisol levels can disrupt normal gut contractions, contributing to irregular bowel movements, including episodes of diarrhea and constipation [94]. Furthermore, cortisol increases visceral sensitivity by enhancing the pain transmission pathways from the intestines to the brain, which exacerbates abdominal pain and discomfort. This effect often correlates with the levels of stress and emotional state [95]. Additionally, cortisol modulates inflammatory and immune responses within the gut, which can affect motility and sensitivity. While IBS is primarily classified as a functional disorder, chronic stress and elevated cortisol levels may intensify symptoms by disrupting normal immune responses and inflammation [96].

The management of IBS focuses on alleviating symptoms and improving the overall quality of life. Treatment strategies often include dietary modifications, such as avoiding known trigger foods or implementing a low-FODMAP diet, which reduces fermentable carbohydrates known to worsen symptoms. Pharmacological interventions may involve medications such as antispasmodics or laxatives, while psychological therapies like cognitive behavioral therapy (CBT) or relaxation techniques can be beneficial in managing stress and improving coping mechanisms [97,98]. A comprehensive understanding of these intricate interactions highlights the importance of addressing stress, cortisol regulation, and gut health in the treatment of IBS. Developing personalized therapeutic strategies that consider these factors is crucial for effective symptom management and improving the well-being of individuals with this complex gastrointestinal disorder [99].

Crohn’s disease is a chronic inflammatory bowel disease that can affect any segment of the gastrointestinal (GI) tract, although it most commonly involves the terminal ileum and the beginning of the colon. The pathogenesis of Crohn’s disease is complex and involves a combination of genetic, environmental, and immunological factors [100]. Genetic predisposition plays a significant role, with mutations in the NOD2/CARD15 gene affecting the immune system’s ability to respond appropriately to microbial antigens, thereby contributing to the disease [101]. Environmental factors, such as smoking and diet, also influence disease development; smoking exacerbates the disease and is a known risk factor, while dietary patterns may affect gut microbiota and immune function [102]. The disease is characterized by an exaggerated immune response to intestinal microbiota, leading to chronic inflammation. This inflammation involves both innate and adaptive immune responses, with the infiltration of inflammatory cells such as T lymphocytes and macrophages and the production of proinflammatory cytokines including TNF-α, IL-1, and IL-6 [103]. This immune dysregulation results in transmural inflammation, causing ulcerations, strictures, and fistulas within the intestinal wall [102,103]. The altered gut microbiota, or dysbiosis, further complicates the disease by influencing immune responses and mucosal permeability, leading to bacterial translocation and persistent inflammation [104]. Clinically, Crohn’s disease presents with symptoms such as persistent diarrhea, abdominal pain, rectal bleeding, weight loss, and fatigue, with complications that may include bowel strictures, fistulas, and an increased risk of colorectal cancer [99]. Treatment aims to induce and maintain remission through pharmacological therapies like corticosteroids, immunosuppressants, and biologics, as well as surgical interventions to manage complications and resect damaged bowel segments [105].

Ulcerative colitis (UC) is a chronic inflammatory bowel disease that primarily affects the colon and rectum, characterized by the recurrent inflammation and ulceration of the mucosal lining of the colon. The disease typically begins in the rectum and may extend continuously to involve varying lengths of the colon, often starting at the rectum and spreading proximally. The inflammation in UC is confined to the mucosa and submucosa, leading to a range of symptoms including abdominal pain, diarrhea (often bloody), an urgent need to defecate, and weight loss [106]. The pathophysiology of ulcerative colitis involves a complex interplay between genetic, environmental, and immunological factors. Although the exact cause remains unclear, it is believed that an abnormal immune response, potentially triggered by genetic predisposition and environmental factors, including changes in the gut microbiota, plays a central role in the disease’s development. In UC, the immune system mistakenly targets the gut’s own tissues, leading to the chronic inflammation and ulceration of the colon’s lining. This persistent inflammation disrupts the normal function of the colon, causing symptoms such as diarrhea and abdominal discomfort. The complications of UC can include anemia due to chronic bleeding, dehydration, and nutritional deficiencies resulting from impaired absorption. In severe cases, the disease can lead to potentially life-threatening complications such as toxic megacolon or colorectal cancer. Management strategies for ulcerative colitis focus on achieving and maintaining the remission of symptoms. This typically involves the use of medications that suppress inflammation, such as corticosteroids, immunomodulators, or biologic therapies. Dietary modifications may also be recommended to alleviate symptoms, and in cases where medication and diet are insufficient, surgical options such as colectomy—the removal of the colon—may be necessary to control symptoms and improve the quality of life [107,108].

Stress and the associated hormone cortisol can significantly impact the course and severity of both Crohn’s disease and ulcerative colitis. Stress, whether acute or chronic, triggers a cascade of physiological responses that can exacerbate inflammation and alter the gut microbiota, both of which are central to the pathogenesis of IBD [109]. As mentioned before, cortisol, the primary glucocorticoid hormone involved in stress responses, plays a complex role in modulating immune function and inflammation [110]. In individuals with IBD, the dysregulation of the HPA axis—responsible for cortisol production and regulation—can lead to abnormal cortisol levels [109]. Elevated cortisol levels during acute stress responses can initially suppress inflammation through anti-inflammatory effects, potentially offering transient relief. However, chronic stress can lead to sustained HPA axis activation and subsequent cortisol resistance, where the anti-inflammatory effects diminish [14]. This dysregulation can exacerbate inflammation in the gastrointestinal tract, leading to increased disease activity and symptoms in both Crohn’s disease and ulcerative colitis patients. Moreover, stress can also influence gut permeability, alter gut microbiota composition, and disrupt intestinal barrier function, further aggravating the inflammatory milieu in the gut. Increased intestinal permeability is a critical factor in the pathogenesis of IBD, exacerbated by chronic stress and elevated cortisol levels [111]. This disruption of the gut barrier contributes to systemic inflammation and metabolic dysregulation, further complicating the clinical management of IBD [111]. Ongoing research is actively exploring the development of novel biomarkers for intestinal permeability, aiming to provide new and non-invasive methods for better assessing and managing IBD. A current study is focused on validating these biomarkers, which could significantly enhance our ability to diagnose and tailor treatments for patients with active IBD [112]. The effective management of IBD thus requires not only controlling inflammation through medical therapies but also addressing stress management strategies to potentially mitigate the impact of stress-induced exacerbations on disease progression and the quality of life for patients [113,114].

## 8. Neurobiological Implications

Chronic stress and persistently elevated cortisol levels have profound effects on neurobiology, contributing to neurodegeneration, neuroinflammation, and various mental health issues, alongside impairments in cognitive functions [14].

### 8.1. Neurodegeneration

Elevated cortisol levels can lead to neurodegenerative changes in the brain. Cortisol, acting through its glucocorticoid receptors, affects neuronal survival, growth, and differentiation. Prolonged exposure to high cortisol levels is associated with neuronal atrophy, particularly in the hippocampus, which is crucial for learning and memory [115]. This atrophy is believed to result from cortisol-induced excitotoxicity and oxidative stress, contributing to the development of neurodegenerative diseases such as Alzheimer’s disease and other forms of dementia. These conditions are characterized by progressive cognitive decline and memory loss, often linked to the accumulation of neurofibrillary tangles and amyloid-beta plaques in the brain—processes that cortisol may exacerbate by affecting neuronal health and function.

Additionally, cortisol can influence neurodegeneration by modulating inflammatory processes in the brain. Elevated cortisol levels can promote neuroinflammation by affecting microglial activity, which is the activity of the brain’s primary immune cells. This neuroinflammation can lead to neuronal damage and worsen neurodegenerative processes. Furthermore, cortisol’s impact on brain glucose metabolism may strain neuronal cells and increase oxidative stress, thereby aggravating neurodegeneration. These complex mechanisms highlight cortisol’s multifaceted role in the pathophysiology of neurodegenerative diseases [116,117].

### 8.2. Neuroinflammation

Cortisol has a specific role in regulating neuroinflammation within the brain. It directly affects microglia, the primary immune cells of the brain, which play a crucial role in neuroinflammatory processes. Cortisol reduces microglial activation and their ability to produce and release proinflammatory cytokines such as IL-1, IL-6, and TNF-α. Simultaneously, cortisol promotes the production of anti-inflammatory cytokines such as IL-10 and TGF-β. These anti-inflammatory cytokines help reduce neuroinflammation and support tissue regeneration following stress or injury. This dual effect of cortisol on neuroinflammation underscores its complex role in maintaining brain health during chronic stress and prolonged exposure. Additionally, cortisol modulates neuroinflammation by regulating other factors such as neurotransmitters and glucose metabolism in the brain. Elevated cortisol levels can impact neurotransmitter systems crucial for mood regulation and stress response, including serotonin, dopamine, and norepinephrine. Furthermore, cortisol’s influence on brain glucose metabolism may affect cellular energy dynamics and their role in managing neuroinflammatory processes. Therefore, cortisol not only directly affects microglial activity and cytokine production but also indirectly modulates neuroinflammation through these additional mechanisms [118,119].

### 8.3. Overall Effect on Mental Health

Elevated cortisol levels significantly impact mental health, particularly in conditions like depression and anxiety. Cortisol, a stress hormone, influences neurotransmitter systems crucial for mood regulation, such as serotonin and dopamine. The chronic elevation of cortisol can disrupt the balance of these neurotransmitters, contributing to the development and exacerbation of mood disorders [120].

In depression, prolonged exposure to high cortisol levels has been linked to changes in brain structure and function. Cortisol can lead to neuronal atrophy, particularly in the hippocampus, a region vital for mood regulation and memory. This atrophy may impair cognitive functions and exacerbate depressive symptoms, including persistent sadness, loss of interest in activities, and sleep disturbances [121]. Studies have shown that MDD, affecting approximately 12% of individuals in the Western world, is closely linked with both acute and chronic pain and stress conditions [122].

Anxiety disorders, similarly, are affected by cortisol’s role in stress response. Elevated cortisol levels can heighten sensitivity to stressors and perpetuate a cycle of anxiety. Cortisol modulates the activity of the amygdala, a brain region involved in emotional responses and fear processing. Dysregulation in this area can lead to increased anxiety symptoms, such as excessive worry, restlessness, and physiological responses like increased heart rate and sweating [123,124]. Furthermore, chronic stress and elevated cortisol levels impair the brain’s ability to adapt and recover from stress, exacerbating both depression and anxiety. Sleep disturbances, common in both conditions, can further disrupt cortisol rhythms, creating a feedback loop that perpetuates mental health challenges [120].

The effective management of cortisol levels through stress reduction techniques, lifestyle modifications, and, in some cases, pharmacological interventions can play a crucial role in mitigating the impact of cortisol on mood disorders. Understanding these mechanisms underscores the importance of holistic approaches to mental health care, addressing both psychological and physiological factors involved in depression and anxiety [14].

### 8.4. Cognitive Functions

Cortisol, often referred to as the “stress hormone”, significantly affects cognitive functions. During acute stress, elevated cortisol levels can temporarily enhance memory, attention, and concentration, allowing for quicker and more efficient problem-solving. However, prolonged elevated cortisol levels can damage the hippocampus, the brain region critical for forming new memories and learning, leading to difficulties with memory and a reduced capacity to acquire new information [125].

Chronic stress can also impair attention, increase concentration problems, and negatively affect executive functions such as planning, decision-making, and impulse control due to damage to the prefrontal cortex [126]. Additionally, elevated cortisol levels can reduce the volume of the amygdala, a region essential for emotional regulation, leading to increased stress sensitivity and diminished emotional control, which may result in anxiety and depression [127].

Extended exposure to high cortisol levels contributes to neurodegenerative processes in the brain, such as neuronal damage in the hippocampus and prefrontal cortex, leading to cognitive decline and a heightened risk of diseases like Alzheimer’s disease [128]. Chronic stress and sustained high cortisol levels also reduce neuroplasticity, impairing the brain’s ability to recover from injuries and adapt to new information and experiences. While cortisol is crucial for normal body function, balance is essential—short-term elevation can be beneficial, but long-term elevation can adversely affect cognitive functions and overall brain health [2].

## 9. Clinical Implications and Novel Therapies

Chronic stress, which leads to elevated cortisol levels, has a profound impact on IBD by affecting gut microbiota and immune function [129]. Elevated cortisol can disrupt the balance of gut microbiota, leading to dysbiosis, which is characterized by a decrease in beneficial bacteria and an increase in harmful microorganisms [130]. This microbial imbalance contributes to the exacerbation of inflammation and can aggravate IBD symptoms. Cortisol-induced dysbiosis may also impair the gut barrier function, making the intestinal lining more permeable and facilitating the translocation of pathogens and toxins into the bloodstream, further exacerbating inflammatory responses [131].

In clinical practice, addressing these effects involves integrating stress management into the treatment regimen for IBD patients. Therapeutic strategies that target cortisol levels and improve gut health are essential [132]. For instance, interventions such as probiotics and prebiotics can help restore microbial balance and strengthen the gut barrier, mitigating some of the negative effects of chronic stress on gut health. Probiotics, such as the Lactobacillus and Bifidobacterium strains, can help replenish beneficial bacteria and enhance mucosal immunity, while prebiotics like inulin can promote the growth of these beneficial microorganisms [133].

Novel therapies include the use of cortisol analogs, which are synthetic compounds designed to mimic the effects of natural cortisol while offering targeted therapeutic benefits with potentially improved safety profiles. Cortisol analogs work by binding to glucocorticoid receptors, regulating the expression of genes involved in inflammation and immune response. Unlike traditional corticosteroids, which can have broad and sometimes adverse systemic effects, cortisol analogs are engineered to target specific pathways more selectively [134]. For example, Selective Glucocorticoid Receptor Agonists (SEGRAs) activate receptors involved in anti-inflammatory responses while minimizing effects on glucose metabolism and bone density [135].

Dual-action cortisol analogs, which combine glucocorticoid activity with additional properties like mineralocorticoid receptor antagonism, help manage inflammation while counteracting some adverse effects related to fluid retention and electrolyte imbalances. Modified-release formulations of cortisol analogs allow for controlled and sustained drug release, mimicking the natural circadian rhythm of cortisol secretion. This approach helps maintain therapeutic levels throughout the day while reducing peak-related side effects [136].

In the context of IBD, cortisol analogs offer several advantages: they provide targeted inflammation control with reduced systemic exposure, potentially leading to fewer side effects compared to traditional corticosteroids. Additionally, the ability to tailor therapy based on the specific effects of different cortisol analogs allows for more personalized treatment strategies. Monitoring the biomarkers of inflammation and cortisol activity can aid in adjusting dosages and selecting the most appropriate analog for individual patients [137].

Ongoing research is focused on optimizing these analogs to enhance their efficacy and safety profiles, with studies exploring their impact on gut microbiota, mucosal immunity, and overall patient health. As our understanding of cortisol’s role in stress and inflammation evolves, new and refined cortisol analogs may emerge, offering even greater benefits for managing chronic stress-related conditions.

## 10. Cortisol Analogs in IBD Treatment

Several cortisol analogs are widely used in the management of IBD, each with unique pharmacokinetic properties, efficacy profiles, and side effect considerations. The most commonly used corticosteroids include prednisone, prednisolone, hydrocortisone, budesonide, methylprednisolone, and dexamethasone [138].

Prednisone and prednisolone are among the most frequently prescribed corticosteroids for managing acute exacerbations of IBD, including Crohn’s disease and ulcerative colitis. Prednisone is a prodrug that undergoes conversion to its active form, prednisolone, in the liver. Both corticosteroids are highly effective in controlling inflammation during severe flare-ups and are often utilized as initial treatments when first-line therapies, such as 5-ASA (5-aminosalicylic acid) or immunomodulators, fail to achieve adequate symptom control. Prednisolone is particularly valued for its strong anti-inflammatory effects and is frequently incorporated into treatment regimens for patients experiencing active inflammation that does not respond to other medications [139]. Despite their effectiveness, the long-term use of prednisone and prednisolone is associated with significant side effects, which can limit their prolonged use. These side effects include osteoporosis, which increases the risk of fractures; hyperglycemia, which can lead to diabetes or worsen existing diabetes; and a heightened susceptibility to infections due to their immunosuppressive effects. Additionally, chronic use can result in adrenal suppression, necessitating the careful management and gradual tapering of the medication to avoid withdrawal symptoms. Therefore, while these corticosteroids are invaluable for managing acute IBD flare-ups, their potential for adverse effects often necessitates a careful monitoring and consideration of alternative therapies for long-term management [140].

Hydrocortisone is frequently used in the management of severe cases of IBD, particularly when patients are hospitalized and require rapid symptom control. Its intravenous administration allows for a swift onset of action, which is crucial for managing acute exacerbations of IBD. Hydrocortisone is effective in quickly reducing systemic inflammation and can be crucial for stabilizing patients in critical situations [138]. For outpatient management or less severe cases, hydrocortisone may be administered orally. Despite its effectiveness, hydrocortisone is associated with several systemic side effects due to its potent glucocorticoid activity. These side effects include fluid retention, which can lead to hypertension and edema, glucose intolerance that may progress to diabetes, and an increased risk of infections due to immune suppression [141]. The potential for severe side effects, such as adrenal suppression and long-term metabolic complications, limits its use to short-term or emergency situations. As a result, hydrocortisone is generally not recommended for prolonged therapy and is reserved for acute disease management [142].

Budesonide, on the other hand, is a synthetic corticosteroid known for its high topical activity and low systemic bioavailability. Its extensive first-pass metabolism in the liver results in minimal systemic absorption, which reduces the risk of systemic side effects compared to other corticosteroids. Budesonide is particularly effective for treating mild-to-moderate Crohn’s disease, especially when inflammation is localized to the ileum or ascending colon. It is typically used in cases where 5-ASA compounds are inadequate or as part of a step-down approach from systemic corticosteroids. The low systemic exposure of budesonide makes it a favorable option for long-term management, as it minimizes risks such as osteoporosis, hypertension, and hyperglycemia. However, its efficacy in severe cases of IBD may be less robust compared to prednisone or prednisolone, and it might not be sufficient for managing extensive or refractory disease. Despite its advantages, regular monitoring is necessary to ensure that it effectively manages disease symptoms without significant side effects [143,144].

Methylprednisolone is a potent corticosteroid similar to prednisolone but with a stronger anti-inflammatory effect. It is often used for conditions that require more intensive anti-inflammatory treatment. In the context of IBD, methylprednisolone is utilized for severe cases or when a rapid and powerful response is necessary. It is available in various formulations, including oral tablets, intravenous injections, and as a high-dose infusion for acute management. Methylprednisolone’s higher potency can provide a quicker relief of inflammation and symptoms, making it suitable for patients who do not respond adequately to prednisolone. However, its use is accompanied by significant potential side effects, including adrenal suppression, increased susceptibility to infections, and metabolic disturbances such as weight gain and glucose intolerance. Long-term use can exacerbate side effects similar to those seen with other corticosteroids, requiring careful dosing and monitoring to mitigate risks [145].

Dexamethasone is a highly potent corticosteroid with a long half-life, making it particularly effective for managing severe or refractory cases of IBD. Its strong anti-inflammatory properties and extended duration of action allow for the effective control of persistent inflammation, especially in cases where other corticosteroids have proven insufficient. Dexamethasone can be administered orally, intravenously, or intramuscularly, depending on the severity of the condition and the clinical situation. While it provides significant therapeutic benefits in severe cases, its potency is associated with a higher risk of serious side effects. These include muscle wasting, significant metabolic disturbances such as severe hyperglycemia and dyslipidemia, and adrenal suppression. The potential for such severe side effects necessitates that dexamethasone be used cautiously and typically only in patients who require aggressive treatment. Regular monitoring and careful management are essential to balance its therapeutic benefits with the risk of adverse effects

Several clinical studies have compared the effectiveness of these corticosteroids in IBD treatment. For example, trials have demonstrated that prednisone is more effective than budesonide in inducing remission in moderate-to-severe Crohn’s disease but at the cost of more severe side effects. In contrast, budesonide is associated with fewer side effects and is better tolerated in long-term use but may be less effective in maintaining remission in severe cases. Hydrocortisone remains a mainstay in the acute management of severe IBD due to its potent effects, but its use is typically limited to short-term therapy due to the risk of significant adverse effects.

Each corticosteroid has its specific use cases and potential side effects, making the choice of medication dependent on the severity of the disease, the patient’s medical history, and the need for either short-term control or long-term management. The selection and dosing of corticosteroids must balance therapeutic efficacy with the risk of adverse effects to ensure optimal patient outcomes [146,147].

## 11. Conclusions

In conclusion, this paper has explored the intricate interplay between chronic stress, metabolic dysregulation, and IBDs, emphasizing their neurobiological implications. Chronic stress, characterized by the prolonged activation of the HPA axis and elevated cortisol levels, contributes significantly to the exacerbation of IBD symptoms and progression of the disease. The dysregulation of cortisol not only impacts gut inflammation and immune responses but also affects neurobiological processes, leading to neurodegeneration, neuroinflammation, and mental health disorders such as depression and anxiety.

Metabolically, the disruption caused by chronic stress alters gut microbiota composition, increases intestinal permeability, and enhances inflammatory cytokine production, thereby aggravating IBD pathology. These findings underscore the importance of comprehensive management strategies that include stress reduction techniques, dietary modifications, and targeted pharmacological interventions to mitigate cortisol’s detrimental effects on both gastrointestinal and neurological health.

Moving forward, further research is warranted to elucidate the specific mechanisms linking chronic stress, metabolic dysregulation, and neurobiological outcomes in IBDs. Additionally, exploring novel therapeutic approaches that integrate stress management with conventional treatments could potentially improve clinical outcomes and enhance the quality of life for individuals affected by these complex diseases.
